# EMOTION REGULATION AND POSITIVE AFFECT IN SPOUSAL DEMENTIA CARE DYADS

**DOI:** 10.1093/geroni/igac059.1575

**Published:** 2022-12-20

**Authors:** Amanda Piechota, Joshua Novak, Thi Vu, Joan Monin

**Affiliations:** Yale School of Public Health, New Haven, Connecticut, United States; Auburn University, Auburn, Alabama, United States; Yale University, New Haven, Connecticut, United States; Yale School of Public Health, New Haven, Connecticut, United States

## Abstract

The early stages of dementia can be a time of stress and uncertainty for spouses, yet little attention is paid to positive experiences. It is important to understand whether there are individual differences in emotion regulation that impact the positive affect of the individual and the partner. Drawing from interdependence theory, we hypothesized that one spouse’s greater difficulty regulating emotions would be associated with their spouses lower positive emotions over time. We used self-report data from an intervention study with three assessment points (baseline, two weeks, and three months) of 45 older adult married couples (N=90) where one spouse has early-stage dementia. Both partners completed the Difficulties in Emotion Regulation Scale and the Positive and Negative Affect Scale. Results from the longitudinal actor partner interdependence model showed that when care partners were high in difficulty regulating emotions, their partner’s positive affect decreased (β = -0.343, p <.01), controlling for intervention arm and covariates.

